# Setting research priorities on multiple micronutrient supplementation in pregnancy

**DOI:** 10.1111/nyas.14267

**Published:** 2019-11-06

**Authors:** Filomena Gomes, Megan W. Bourassa, Seth Adu-Afarwuah, Clayton Ajello, Zulfiqar A. Bhutta, Robert Black, Elisabete Catarino, Ranadip Chowdhury, Nita Dalmiya, Pratibha Dwarkanath, Reina Engle-Stone, Alison D. Gernand, Sophie Goudet, John Hoddinott, Pernille Kæstel, Mari S. Manger, Christine M. McDonald, Saurabh Mehta, Sophie E. Moore, Lynnette M. Neufeld, Saskia Osendarp, Prema Ramachandran, Kathleen M. Rasmussen, Christine Stewart, Christopher Sudfeld, Keith West, Gilles Bergeron

**Affiliations:** 1 The New York Academy of Sciences, New York, New York; 2 University of Ghana, Legon, Ghana; 3 Vitamin Angels, Santa Barbara, California; 4 Centre for Global Child Health, the Hospital for Sick Children, Toronto, Ontario, Canada; 5 Center of Excellence in Women and Child Health, the Aga Khan University, Karachi, Pakistan; 6 Johns Hopkins Bloomberg School of Public Health, Baltimore, Maryland; 7 Independent Consultant, Maputo, Mozambique; 8 Centre for Health Research and Development, Society for Applied Studies, New Delhi, India; 9 UNICEF, New York, New York; 10 Division of Nutrition, St. John’s Research Institute, Bangalore, India; 11 UC Davis Department of Nutrition, Davis, California; 12 Pennsylvania State University, University Park, Pennsylvania; 13 Loughborough University, Loughborough, England; 14 Division of Nutritional Sciences, Cornell University, Ithaca, New York; 15 Department of Nutrition, Exercise and Sports, University of Copenhagen, Copenhagen, Denmark; 16 IZiNCG, Children’s Hospital Oakland Research Institute Oakland, California; 17 Department of Women & Children’s Health, King’s College London, London, United Kingdom; 18 Global Alliance for Improved Nutrition, Geneva, Switzerland; 19 Micronutrient Forum, Washington, DC; 20 Nutrition Foundation of India, New Delhi, India; 21 Harvard T.H. Chan School of Public Health, Boston, Massachusetts

**Keywords:** pregnancy, micronutrients, supplementation, research priorities, low- and middle-income countries

## Abstract

Prenatal micronutrient deficiencies are associated with negative maternal and birth outcomes. Multiple micronutrient supplementation (MMS) during pregnancy is a cost-effective intervention to reduce these adverse outcomes. However, important knowledge gaps remain in the implementation of MMS interventions. The Child Health and Nutrition Research Initiative (CHNRI) methodology was applied to inform the direction of research and investments needed to support the implementation of MMS interventions for pregnant women in low- and middle-income countries (LMIC). Following CHNRI methodology guidelines, a group of international experts in nutrition and maternal health provided and ranked the research questions that most urgently need to be resolved for prenatal MMS interventions to be successfully implemented. Seventy-three research questions were received, analyzed, and reorganized, resulting in 35 consolidated research questions. These were scored against four criteria, yielding a priority ranking where the top 10 research options focused on strategies to increase antenatal care attendance and MMS adherence, methods needed to identify populations more likely to benefit from MMS interventions and some discovery issues (e.g., potential benefit of extending MMS through lactation). This exercise prioritized 35 discrete research questions that merit serious consideration for the potential of MMS during pregnancy to be optimized in LMIC.

## Introduction

Adequate nutrition is important throughout the life cycle but is particularly important during pregnancy, to support both maternal health and fetal development. Many micronutrients have critical roles during this life stage (especially vitamins A, B_6_, B^9^, B^12^, C, D, and E, and mineralsiron, zinc, iodine, copper, and selenium^1^) for which the recommended intakes may increase by up to 50% to accommodate the higher maternal, placental, and fetal demands. These increased nutritional demands of pregnancy, in combination with the preexisting nutritional deficiencies among undernourished (and/or the even higher nutritional demands for adolescent) pregnant women, may put their health and that of their offspring at risk.^2^ Maternal micronutrient malnutrition is associated with low birth weight (LBW) (*<* 2500 g), preterm birth (*<* 37 weeks), being born small-for-gestational-age (SGA), and perinatal and maternal mortality, among other pregnancy-related adverse outcomes.^1^

Prenatal multiple micronutrient supplementation (MMS) provides a good solution to supply those essential nutrients. A series of publications recently put forward by the New York Academy of Sciences (NYAS) presented evidence of the benefits of MMS on maternal and perinatal outcomes (i.e., significant risk reduction of LBW, SGA, preterm birth, and stillbirth^3^), in addition to those provided by iron and folic acid.^2,4–6^ These publications identify populations in low- and middle-income countries(LMIC), where a switch to MMS would be justified, consistent with the WHO Antenatal Care Guidelines,^7^ and would be highly cost-effective.^4^ However, important gaps in knowledge remain in the implementation of MMS in prenatal care programs, which affect the ability of this intervention to achieve optimal performance.

To clarify research needs in solving knowledge gaps in MMS implementation, NYAS, acting on behalf of the recently assembled MMS Technical Advisory Group (MMS-TAG), conducted a research prioritization exercise using the well-established Child Health and Nutrition Research Initiative (CHNRI) methodology.^8^ CHNRI provides a systematic and transparent method for setting priorities in health research^8^ using a rationale, conceptual framework, application guidelines, and strategies to address the needs of various stakeholders.^9^ It has been used mostly for setting research priorities to address global child health issues (e.g., zinc deficiency^9^), as initially designed, but has also been applied outside this field (e.g., dementia^10^), as this method is replicable, simple, democratic, flexible, and adjustable to many different contexts and needs.^11^

The specific aim of this research prioritization exercise was to inform the direction of research and investments needed to support the implementation of MMS interventions for pregnant women in LMIC. The ultimate aim is to reduce the burden associated with micronutrient deficiencies in this vulnerable population group, particularly the adverse pregnancy and birth outcomes.

A first step in the CHNRI method consists of defining the context (population of interest, disease burden, timescale, etc.) and risk preferences associated with this research priority setting exercise (Table 1).

## Methods

As recommended by the guidelines for the implementation of the CHNRI method,^8,11^ the following steps were followed:

1. Selected the project managers for this exercise (F.G., G.B., and M.W.B., from the MMSTAG secretariat at the NYAS) and specified the context and risk management preferences (Table 1).
2. Selected and asked a small group of specialists in nutrition and maternal health (mostly from the MMS-TAG) to contribute research questions believed to be important for the implementation of prenatal MMS interventions in LMIC.
3. Organized a large number of the proposed research questions into four fundamental instruments of health research (i.e., the four domains: “description,” “delivery,” “development,” and “discovery”); removed or combined redundant questions to obtain a consolidated final list of questions. The four domains were defined as:
  - Description: What is the burden of the problem and its determinants, for example, feasible methods to determine if a population has multiple micronutrient deficiencies?
  - Delivery: How can we improve the delivery of existing interventions, for example, how to improve adherence and compliance?
  - Development: How do we improve the efficiency of existing interventions, for example, by modifying the product formulation, reducing costs, and so on?
  - Discovery: Does the research lead to innovation, for example, by demonstrating new benefits in trials or mechanisms of action of MMS?
    To facilitate classification, each domain was, in turn, divided into subdomains, that is, “description” questions were classified under “prevalence” or “assessment”; “delivery” questions were classified under “adherence,” “coverage,” “packaging,” or “training”; “development” questions were classified under “dosage” or “implementation”; and “discovery” questions were classified under “formulation” or “impact.”
4. Developed and discussed the criteria for setting health research priorities. Four proposed criteria were later used to rate each research question based on whether the research question was:
  - Answerable: Is it feasible to answer this research question within 5–10 years?
  - Impactful: Are the results from this research likely to inform future practice and/or policy on MMS during pregnancy?
  - Effective: Will the research effectively improve maternal and birth outcomes?
  - Equitable: Will the results of the research help enable the benefits of MMS to reach the poorest and currently underserved women?
5. Constructed an evaluation template and sent it to a broader group of specialists (including antenatal care (ANC) managers and nongovernmental organizations operating in this space, identified by the MMS-TAG) to score the final list of research questions against each priority-setting criterion, and to attribute weights to each of these criteria. Attributing weights to each criterion (from 0% to 100%) allowed each participant to give some criteria more importance over the others.

The Likert scale used to answer each of the four criteria questions and the score attributed to each answer was:

- Highlylikely(score:1)
- Somewhat likely (score: 0.75)
- Neutral, neither likely, or unlikely (score: 0.5)
- Somewhat unlikely (score: 0.25)
- Highlyunlikely(score:0)
- Do not know (no score; coded as a missing answer)

6 Compiled the data, calculating the final unweighted and weighted research priority scores (RPSs) for each research question, and assigned ranks. To calculate the final unweighted RPS, the average of the scores for each of the four criteria (from all the participants) was computed, followed by the calculation of the mean of these four criteria scores (i.e., all four criteria were given equal weight, equivalent to 25%). For the calculation of the weighted RPS, the average of the priority scores for each of the four criteria (from all the participants) was computed, taking into account the group average weight attributed to each criterion. The weighted RPS was calculated using the following formula (Eq. 1):
  Weighted Research Priority Score
  (1) $$=\frac{W_{a}C_{a}+W_{i}C_{i}+W_{e}C_{e}+W_{q}C_{q}}{W_{a}+W_{i}+W_{e}+W_{q}}$$
  where *C_a_* is the average criterion score for answerable, *C_i_* is the average criterion score for impactful, *C_e_* is the average criterion score for effective, *C_q_* is the average criterion score for equitable, *W_a_* is the average weight for answerable, *W_i_* is the average weight score for impactful, *W_e_* is the average weight score for effective, and *W_q_* is the average weight score for equitable.
  In addition, the average expert agreement (AEA) score, defined as the level of agreement among scorers, was calculated for each of the 35 research questions. The AEA score is the average proportion of scorers who chose the mode (most common score) across the four criteria. The AEA score was calculated as follows (Eq. 2):
  (2) $$\begin{array}{l} \text{AEA}=\frac{1}{4}\times \overset{4}{\underset{q=1}{\sum }}\\ \left(\frac{N\left(\text{Scorers who contributed the most common response}\right)}{N\left(\text{scorers}\right)}\right) \end{array}$$
  where *q* is a question that experts are being asked to evaluate, ranging from 1 to 35.

**Table 1:** Context of the research prioritization exercise on multiple micronutrient supplementation in pregnancy

Area	CHNRI guideline^8^	Context of the prioritization exercise
Population of interest	Whose health issues are being addressed?	Fetus and infants 0–11 months old Pregnant women
The disease burden of interest	What is known about the burden of disease, disability, and death that will be addressed by supported health research?	Anemia affects 31.6% of pregnant women in LMIC; globally, 63.2% of WRA are vitamin D deficient, 41.4% are zinc deficient, 22.7% are folate deficient, and 15.9% are vitamin A deficient^2^ Adverse pregnancy and birth outcomes associated with maternal micronutrient deficiencies include: - LBW: 14.6% of all live births globally with 91% from LMIC^12^ - SGA: 19.3% (23.3 million) of all live births in LMIC (28% in South Asia); 606,500 neonatal deaths attributable to SGA^2^ - Preterm births: 10.6% (14.84 million) of live births globally; *>* 80% from Asia and Sub-Saharan Africa^2^
Geographic limits	Spatial boundaries (global, regional, national, etc.)	LMIC with evidence of poor pregnancy and birth outcomes
		Research that focuses on subnational, national, regional, or global levels
Timescale	Level of urgency, that is, in how many years are the first results of the proposed research expected	Achieve measurable results within 5–10 years
Preferred style of investing with respect to risk	Investment strategy in health research with respect to risk preferences: should most of the funding support a single (or a few) expensive high-risk research ideas (e.g., vaccine development), or be balanced and diversified between many research options, which show different levels of risk and feasibility?	Research will be diversified across countries that show a high prevalence of micronutrient deficiencies among pregnant women and/or high rates of adverse pregnancy and birth outcomes, which will have different levels of risk and feasibility

CHRNI, Child Health and Nutrition Research Initiative; LBW, low birth weight; LMIC, low- and middle-income countries; SGA, small-for-gestational-age; WRA, women of reproductive age.

## Results

This prioritization exercise was conducted between April and June 2019 with a group of international experts in nutrition and maternal health, who provided and ranked the most urgent gaps in knowledge for the successful implementation of prenatal MMS interventions. This group included participants based on a variety of countries, including Bangladesh, Canada, Denmark, Ghana, India, Italy, Kenya, Mozambique, Switzerland, Thailand, the United Kingdom, and the United States of America.

**Figure 1:**
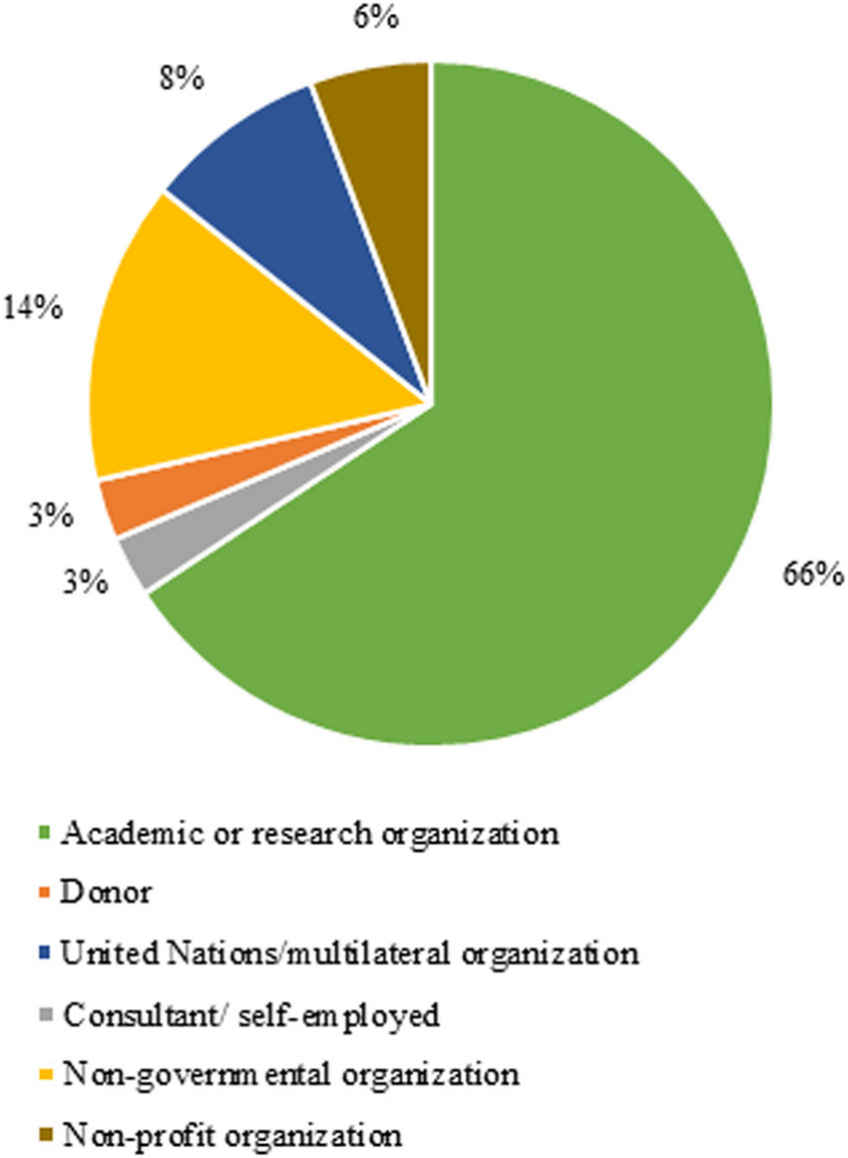
Types of organizations where the 35 individuals who participated in the evaluation exercise work.

The initial small group of 25 experts submitted a total of 73 research questions, which were analyzed and reorganized (e.g., by eliminating or combining redundant questions), resulting in a final number of 35 research questions. The evaluation exercise was then sent to a larger group of experts (*n* = 87), who were invited to score each of the 35 research questions against the four evaluation criteria. Thirty-five participants completed the evaluation exercise and the summary of the results is presented in Tables 2–4. These 35 participants work in a wide range of organizations, as exemplified in Figure 1, including academic or research, nongovernmental, the United Nations or multilateral, and nonprofit organizations, donors, and also work as consultants or are self-employed.

**Table 2:** Research questions ranked according to the final unweighted research priority score (RPS)

Research priority score, unweighted (%)	Rank	AEA score	Question	Domain	Subdomain
83.2	1	0.47	What strategies (cash transfers, easier ANC access, free MMS, pharmacy vouchers, quality service delivery, mass media, social and behavior change communication interventions, SMS text messages, etc.) can best increase ANC attendance and adherence to MMS, including in hard-to-reach populations?	Delivery	Coverage
82.8	2	0.50	What limited set of biomarkers of nutritional status (e.g., hemoglobin) and their cutoffs can be used to identify populations that will benefit from prenatal MMS?	Description	Assessment
81.1	3	0.53	If MMS were continued through lactation, are there additional benefits for the mother and child (e.g., reduced mortality, infection, improved development, etc.)?	Discovery	Impact
80.8	4	0.49	Can community workers help identify pregnancies in the first trimester and facilitate timely ANC attendance that leads to an earlier initiation of MMS?	Delivery	Coverage
79.0	5	0.43	What is the burden of micronutrient deficiencies among pregnant women?	Description	Prevalence
78.5	6	0.46	What field-friendly methods can be used to assess multiple micronutrient deficiencies among pregnant women? (contrast all methods along cost-effectiveness, invasiveness, and training requirements)	Description	Assessment
76.0	7	0.42	Which essential micronutrients (e.g., biomarkers or intake) beyond iron should be routinely monitored for pregnant women?	Description	Assessment
75.2	8	0.39	Are MMS in pregnancy effective in women with low intakes of energy and protein?	Discovery	Impact
74.4	9	0.49	What are the most effective counseling strategies about the benefits of MMS in pregnancy that lead to increased adherence to the MMS regimen?	Delivery	Adherence
73.6	10	0.42	What MMS dosage (timing and duration) should be recommended in prepregnancy and pregnancy to achieve maximum adherence and benefits on outcomes?	Development	Implementation
73.0	11	0.50	Can human-centered design principles (focused on the needs, contexts, behaviors, and emotions of the people) be used to increase the effectiveness of behavior-change programs and increase adherence to prenatal MMS?	Delivery	Adherence
73.0	12	0.47	How can a policy framework be strengthened within a country to ensure the availability of MMS supplements?	Development	Implementation
72.7	13	0.40	To what extent do MMS benefit maternal health (not just anemia or pregnancy outcomes)?	Discovery	Impact
71.5	14	0.50	What are sufficient and cost-effective training options when switching from IFA to MMS, for example (1) standard onetime in-service training; (2) enhanced training, supervision, and coaching delivered routinely every few weeks for an initial period; and (3) enhanced training plus community engagement and promotion?	Delivery	Training
71.4	15	0.40	What is the optimal dose of iron (30 versus 60 mg) in MMS to achieve maximum benefits on maternal and birth outcomes? Does it vary by context, population prevalence of anemia, and dosage of other nutrients (e.g., vitamin C)?	Development	Dosage
71.3	16	0.42	What is the most cost-effective packaging of MMS (i.e., blister packs or bulk packaging; 30-, 90-, or 180-count bottles, etc.) that will optimize both cost and adherence, without adversely affecting ANC attendance?	Delivery	Packaging
70.9	17	0.43	In pregnant women taking MMS who develop iron deficiency anemia, what is the ideal amount and duration of additional iron supplements?	Development	Dosage
70.2	18	0.49	What data commonly available in national surveys can be used to identify populations that will benefit from prenatal MMS?	Description	Prevalence
70.2	19	0.40	What indicators can be measured through routine health information systems to best monitor program performance in relation to MMS delivery during pregnancy (through ANC contacts)?	Delivery	Coverage
69.7	20	0.42	To what extent do infections blunt the impact of prenatal MMS in preventing anemia?	Discovery	Impact
69.3	21	0.51	What are the predictive risk factors of micronutrient deficiencies among pregnant women?	Description	Prevalence
68.3	22	0.40	Is fortification of food staples or ensuring intake of fortified foods (such as lipid-based nutrient supplements) better than providing MMS at scale, on maternal and birth outcomes?	Discovery	Formulation
68.0	23	0.48	Would pregnancy outcomes be further improved by the addition of calcium to MMS, given WHO recommendations for calcium supplementation during pregnancy to reduce the risk of preeclampsia? How would this affect adherence, costs, and stability (given iron and calcium interaction)?	Discovery	Formulation
67.9	24	0.47	Would outcomes be further improved by the addition of choline to MMS, especially with regard to child development? What would be the cost implications?	Discovery	Formulation
67.7	25	0.50	How can implementation research be most efficientìy conducted (time and cost) to improve adherence to prenatal MMS?	Delivery	Adherence
66.9	26	0.44	What is the effectiveness, in terms of the availability, acceptability, and adherence of public versus private sector MMS distribution?	Delivery	Coverage
66.3	27	0.39	Would birth outcomes be further improved by the addition of n-3 LC-PUFA to MMS, given a recent Cochrane meta-analysis showing reduction in preterm delivery with n-3 LC-PUFA supplementation? What would be the cost implications?	Discovery	Formulation
65.2	28	0.35	Are there subpopulations at risk of adverse outcomes with MMS, such as stillbirths or perinatal asphyxia?	Discovery	Impact
65.1	29	0.40	Would outcomes be further improved by the addition of magnesium to MMS? What would be the implications on adherence and costs?	Discovery	Formulation
64.8	30	0.41	Is selenium deficiency independently associated with prematurity and small-for-gestational-age?	Discovery	Impact
64.3	31	0.37	When compared with UNIMMAP, are there more cost-effective formulations?	Development	Dosage
61.8	32	0.32	What is the most appropriate dosage for each micronutrient, other than iron?	Discovery	Formulation
55.6	33	0.34	How does micronutrient status during early life development relate to adult-onset of noncommunicable diseases?	Description	Prevalence
55.0	34	0.33	Why is MMS more successful in preventing infant mortality in female than in male infants?	Discovery	Impact
52.7	35	0.33	What is the marginal cost and marginal benefit of adding each vitamin/mineral to MMS?	Development	Dosage

Note: The questions are color coded by the type of domain: yellow for “description,” green for “delivery,” orange for “development,” and blue for “discovery.”

AEA, average expert agreement.

Table 2 shows the consolidated list of 35 research questions ranked according to the final unweighted RPS, with the respective domain and subdomain for each question. The questions are color coded by type of domain: yellow for “description,” green for “delivery,” orange for “development,” and blue for “discovery.” The top 10 research options include questions that focused on strategies to increase ANC attendance and MMS adherence, as well as on the parameters and methods needed to identify the populations that are more likely to benefit from prenatal MMS interventions, and some discovery issues (e.g., the potential benefit of extending MMS interventions beyond pregnancy, and during the period of lactation). The AEA ranged from 0.32 to 0.53, out of a possible 1.00.

Table 3 describes the number of research questions allocated to each domain and subdomain, as well as the mean, minimum, and maximum RPSs for each subdomain. The fourth domain “discovery” received a higher number of questions, while the lower number of questions were allocated to the third domain “development.” The higher RPS was observed in the subdomain “assessment” and the lower score was attributed to the subdomain “dosage.”

As described above, participants were also asked to attribute weights to each of the four evaluation criteria. The group average weight attributed to answerable was 32.2%, to impactful was 25.5%, to effective was 25%, and to equitable was 17.1%.

Table S1 (online only) shows the consolidated list of 35 research questions ranked according to the final weighted RPS, and color coded according to the respective domain. The results presented in this table were similar to those presented in Table 2, and the top 10 research options include similar topics between the ranks established by the unweighted and weighted RPSs. Table 4 lists the ranked final number of research questions and allows the comparison between the average unweighted RPS (in black) and the weighted RPS (in red), per each of the four evaluation criteria. This table helps to understand, for instance, that the question ranked overall in the third place (regarding the benefits of MMS supplementation continued through lactation) was considered the most answerable, and the question ranked overall in the first place (covering strategies that best increase ANC attendance and adherence to MMS) was considered the most impactful, independently of the use of weighted or unweighted criteria.

## Discussion

This paper summarizes the research prioritization exercise conducted on the topic of MMS in pregnancy—an exercise that has never been conducted. The NYAS invited a group of international specialists to provide and rank the most urgent gaps in knowledge, focusing particularly on aspects that would improve the delivery and effectiveness of this intervention in LMIC populations. Following the well-established CHNRI methodology, this process prioritized 35 nonredundant research questions that merit serious considerations if the potential of MMS is to be fully realized in LMIC.

**Table 3:** Mean, minimum, and maximum unweighted research priority scores (RPSs) by research subdomain

Domain	*n*	Subdomain	n	Mean RPS (%)	Minimum RPS (%)	Maximum RPS(%)
Description	7	Assessment	3	79.1	76.0	82.8
		Prevalence	4	68.5	55.6	79.0
Delivery	9	Adherence	3	71.7	67.7	74.4
		Coverage	4	75.3	66.9	83.2
		Packaging	1	71.3	-	-
		Training	1	71.5	-	-
Development	6	Dosage	4	64.8	52.7	71.4
		Implementation	2	73.3	73.0	73.6
Discovery	13	Formulation	6	66.2	61.8	68.3
		Impact	7	69.1	55.0	81.1

Note: The questions are color coded by the type of domain: yellow for “description,” green for “delivery,” orange for “development,” and blue for “discovery.”

The RPS varied between 52.7% and 83.2%, which is in line with the range observed in previous research priority exercises that used the CHNRI methodology.^9,10^ The AEA showed that from 32% to 53% of the scorers shared their views on the proposed 35 research questions. This range is lower than the AEA ranges reported in other articles that used the CHNRI methodology; for example, the AEA scores ranged from 54% to 86% on the topic of integrated community case management,^13^ and from 53% to 78% on the topic of dementia.^10^ However, this difference is likely to be caused by a greater number of possible answers to the scoring criteria in the present study. For instance, most of those research priority exercises used a simple ordinal scale (“yes,” “no,” and “undecided”) or a dichotomous statement (“yes,” “no,” and “no answer”), while we used a Likert scale, which has a progressive rating (“highly likely,” “somewhat likely,” “neutral,” “somewhat unlikely,” and “highly unlikely”).

The questions that received the highest priority in this exercise include the use of behavioral change and counseling strategies, and community workers who can increase ANC attendance and adherence to MMS, including in hard-to-reach populations. This is not surprising given that low adherence to prenatal micronutrient supplementation is a major barrier to achieve the full potential benefits of this intervention, even when the coverage of the supplementation program is satisfactory. A study across 22 LMIC found that 83% of all pregnant women had at least one ANC visit and a similar proportion (81%) received IFA tablets, but only 8% consumed at least 180 IFA tablets.^14^

In addition, among the highest ranked topics are questions about the best (field-friendly and cost-effective) indicators and methods needed to identify the populations that are more likely to benefit from prenatal MMS interventions in maternal nutrition programs. This may be justified by the fact that countries interested in adopting MMS interventions may feel a lack of clear guidance regarding the interpretation of the statement: “Countries with a high prevalence of nutritional deficiencies might consider the benefits of MMS on maternal health to outweigh the disadvantages and may choose to give MMS that include iron and folic acid,” included in the 2016 WHO Guidelines for ANC.^7^ Governments may want to better identify higher risk groups and regions where the effectiveness of prevention is likely to be the highest and thus offer the greatest public health return on investment.

**Table 4:** Average unweighted (NW) and weighted (W) research priority scores (RPSs) per evaluation criteria

Question		Rank	Final RPS (%)	Answerable	Impactful	Effective	Equitable
What strategies (cash transfers, easier ANC access, free MMS, pharmacy vouchers, quality service delivery, mass media, social and behavior change communication interventions, SMS text messages, etc.) can best increase ANC attendance and adherence to MMS, including in hard-to-reach populations?	Unweighted	1	83.2	84.3	85.7	82.1	80.7
	Weighted	1	83.5	90.4	86.3	82.1	74.3
What limited set of biomarkers of nutritional status (e.g., hemoglobin) and their cutoffs can be used to identify populations that will benefit from prenatal MMS?	Unweighted	2	82.8	84.4	83.0	81.4	82.1
	Weighted	2	82.9	90.5	83.6	81.4	75.7
If MMS were continued through lactation, are there additional benefits for the mother and child (e.g., reduced mortality, infection, improved development, etc.)?	Unweighted	3	81.1	86.4	81.4	82.1	74.3
	Weighted	3	82.0	92.7	82.0	82.1	68.4
Can community workers help identify pregnancies in the first trimester and facilitate timely ANC attendance that leads to earlier initiation of MMS?	Unweighted	4	80.8	80.7	82.9	82.4	77.3
	Weighted	4	81.1	86.5	83.4	82.4	71.2
What is the burden of micronutrient deficiencies among pregnant women?	Unweighted	5	79.0	81.4	82.1	78.7	73.6
	Weighted	5	79.6	87.3	82.7	78.7	67.8
What field-friendly methods can be used to assess multiple micronutrient deficiencies among pregnant women? (contrast all methods along with cost-effectiveness, invasiveness, and training requirements)	Unweighted	6	78.5	75.7	81.4	76.5	80.5
	Weighted	6	78.2	81.2	82.0	76.5	74.1
Which essential micronutrients (e.g., biomarkers or intake) beyond iron should be routinely monitored for pregnant women?	Unweighted	7	76.0	83.3	75.9	72.0	72.7
	Weighted	7	76.8	89.3	76.4	72.0	67.0
Are MMS in pregnancy effective in women with low intakes of energy and protein?	Unweighted	8	75.2	75.7	72.1	75.7	77.2
	Weighted	9	75.0	81.2	72.6	75.7	71.1
What are the most effective counseling strategies about the benefits of MMS in pregnancy that lead to increased adherence to the MMS regimen?	Unweighted	9	74.4	79.3	73.6	75.7	69.1
	Weighted	8	75.2	85.0	74.1	75.7	63.7
What MMS dosage (timing and duration) should be recommended in prepregnancy and pregnancy to achieve maximum adherence and benefits on outcomes?	Unweighted	10	73.6	70.6	80.0	80.0	64.0
	Weighted	10	74.2	75.7	80.6	80.0	58.9
Can human-centered design principles (focused on the needs, contexts, behaviors, and emotions of the people) be used to increase the effectiveness of behavior-change programs and increase adherence to prenatal MMS?	Unweighted	11	73.0	80.6	70.2	72.6	68.5
	Weighted	11	73.9	86.5	70.7	72.6	63.1
How can a policy framework be strengthened within a country to ensure the availability of MMS supplements?	Unweighted	12	73.0	73.5	77.9	69.1	71.3
	Weighted	13	73.2	78.8	78.5	69.1	65.7
To what extent do MMS benefit maternal health (not just anemia or pregnancy outcomes)?	Unweighted	13	72.7	75.7	72.1	74.3	68.8
	Weighted	12	73.2	81.2	72.6	74.3	63.3
What are the sufficient and cost-effective training options when switching from IFA to MMS, for example (1) standard one-time in-service training; (2) enhanced training, supervision and coaching delivered routinely every few weeks for an initial period; and (3) enhanced training plus community engagement and promotion?	Unweighted	14	71.5	80.1	73.5	70.5	62.1
	Weighted	15	72.9	85.9	74.0	70.5	57.2
What is the optimal dose of iron (30 versus 60 mg) in MMS to achieve maximum benefits on maternal and birth outcomes? Does it vary by context, population prevalence of anemia and dosage of other nutrients (e.g., vitamin C)?	Unweighted	15	71.4	72.8	72.8	75.7	64.4
	Weighted	17	72.1	78.0	73.3	75.7	59.3
What is the most cost-effective packaging of MMS (i.e., blister packs or bulk packaging; 30-, 90-, or 180-count bottles, etc.) that will optimize both cost and adherence, without adversely affecting ANC attendance?	Unweighted	16	71.3	86.0	71.3	66.2	61.7
	Weighted	14	73.1	92.2	71.8	66.2	56.8
In pregnant women taking MMS who develop iron deficiency anemia, what is the ideal amount and duration of additional iron supplements?	Unweighted	17	70.9	75.7	73.4	76.5	58.1
	Weighted	16	72.3	81.2	73.9	76.5	53.5
What data commonly available in national surveys can be used to identify populations that will benefit from prenatal MMS?	Unweighted	18	70.2	77.1	70.0	65.4	68.4
	Weighted	18	70.9	82.7	70.5	65.4	63.0
What indicators can be measured through routine health information systems to best monitor program performance in relation to MMS delivery during pregnancy (through ANC contacts)?	Unweighted	19	70.2	74.3	69.9	69.1	67.6
	Weighted	19	70.7	79.6	70.3	69.1	62.3
To what extent do infections blunt the impact of prenatal MMS in preventing anemia?	Unweighted	20	69.7	74.3	70.5	68.9	65.3
	Weighted	20	70.4	79.6	70.9	68.9	60.2
What are the predictive risk factors of micronutrient deficiencies among pregnant women?	Unweighted	21	69.3	75.7	66.2	64.7	70.6
	Weighted	21	69.6	81.2	66.6	64.7	65.0
Is fortification of food staples or ensuring intake of fortified foods (such as lipid-based nutrient supplements) better than providing MMS at scale, on maternal and birth outcomes?	Unweighted	22	68.3	56.4	74.3	74.2	68.4
	Weighted	27	67.5	60.5	74.8	74.2	63.0
Would pregnancy outcomes be further improved by the addition of calcium to MMS, given WHO recommendations for calcium supplementation during pregnancy to reduce the risk of preeclampsia? How would this affect adherence, costs, and stability (given iron and calcium interaction)?	Unweighted	23	68.0	71.2	71.1	69.5	60.2
	Weighted	23	68.9	76.3	71.6	69.5	55.4
Would outcomes be further improved by the addition of choline to MMS, especially with regard to child development? What would be the cost implications?	Unweighted	24	67.9	73.5	65.2	72.7	60.5
	Weighted	22	68.9	78.8	65.6	72.7	55.7
How can implementation research be most efficiently conducted (time and cost) to improve adherence to prenatal MMS?	Unweighted	25	67.7	71.6	72.4	64.7	62.1
	Weighted	25	68.4	76.7	72.9	64.7	57.2
What is the effectiveness, in terms of availability, acceptability, and adherence of public versus private sector MMS distribution?	Unweighted	26	66.9	74.2	67.4	66.4	59.4
	Weighted	26	68.0	79.6	67.9	66.4	54.7
Would birth outcomes be further improved by the addition of n-3 LC-PUFA to MMS, given a recent Cochrane meta-analysis showing a reduction in preterm delivery with n-3 LC-PUFA supplementation? What would be the cost implications?	Unweighted	27	66.3	79.0	68.5	67.5	50.0
	Weighted	24	68.5	84.7	69.0	67.5	46.1
Are there subpopulations at risk of adverse outcomes with MMS, such as stillbirths or perinatal asphyxia?	Unweighted	28	65.2	62.5	67.2	66.9	64.2
	Weighted	30	65.2	67.0	67.7	66.9	59.1
Would outcomes be further improved by the addition of magnesium to MMS? What would be the implications for adherence and costs?	Unweighted	29	65.1	71.0	63.7	69.4	56.5
	Weighted	29	66.2	76.1	64.2	69.4	52.0
Is selenium deficiency independently associated with prematurity and small-for-gestational-age?	Unweighted	30	64.8	78.1	58.1	64.7	58.3
	Weighted	28	66.2	83.8	58.5	64.7	53.7
When compared to UNIMMAP, are there more cost-effective formulations?	Unweighted	31	64.3	66.4	68.2	65.2	57.5
	Weighted	31	65.0	71.2	68.7	65.2	53.0
What is the most appropriate dosage for each micronutrient, other than iron?	Unweighted	32	61.8	57.4	65.4	67.6	56.6
	Weighted	32	61.9	61.5	65.9	67.6	52.1
How does micronutrient status during early life development relate to adult-onset of noncommunicable diseases?	Unweighted	33	55.6	52.1	62.1	55.3	53.0
	Weighted	33	55.6	55.9	62.6	55.3	48.8
Why is MMS more successful in preventing infant mortality in female infants than in male infants?	Unweighted	34	55.0	53.8	50.8	58.3	57.0
	Weighted	34	54.7	57.7	51.1	58.3	52.5
What is the marginal cost and marginal benefit of adding each vitamin/mineral to MMS?	Unweighted	35	52.7	46.2	53.0	56.8	54.8
	Weighted	35	52.1	49.5	53.4	56.8	50.5

The research question that received the lowest score pertains to the marginal cost and benefits of adding each vitamin or mineral to MMS. The group of specialists may have attributed less importance to this question in light of the widely used and well-established UNICEF/WHO/United Nations University International Multiple Micronutrient Preparation “UNIMMAP” formulation,^15^ which has been administered in many of the trials that demonstrated the additional benefits of MMS over IFA^3^ andprovedtobecost-effective.^4,16^ While the addition of a variety of nutrients known to be important during pregnancy (i.e., calcium, magnesium, choline, and n-3 long-chain polyunsaturated fatty acids) was proposed in several research questions listed here, it is possible that a consideration of the time and resources that would be required to answer these questions contributed to their relatively lower RPS.

Overall, it was relatively straightforward to apply the CHNRI methodology to the implementation of MMS interventions for pregnant women in LMCI. Nonetheless, a few challenges were encountered. For instance, the allocation of each research question to the four domains “description,” “delivery,” “development,” and “discovery” proposed by the CHRNI methodology^11^ was not always clear, as some questions did not fit clearly into any domain, while others could belong to more than one domain. In fact, other authors who used the CHNRI method to reduce children’s disease burden^9^ have used other domains, namely “effectiveness,” “affordability, deliverability, and sustainability,” and “new innovations.” Reducing the number of research questions into a consolidated, smaller, and more easily manageable final list of questions also resulted in excessive aggregation of several topics. For example, the question that received the highest RPS (“What strategies (e.g., cash transfers, easier ANC access, free MMS, pharmacy vouchers, quality service delivery, mass media, social and behavior change communication interventions, and SMS text messages) can best increase ANC attendance and adherence to MMS, including in hard-to-reach populations?”) combined a wide variety of different strategies, which may not be easily comparable. Furthermore, the answer to this question may vary from country to country, or from region to region, depending on the service delivery bottlenecks associated with the two proposed outcomes.

A limitation of the current exercise is that respondents were not asked to take into account cost considerations to answer the research questions, except indirectly by the first scoring criterion (where the participant is asked whether it is feasible to answer the research question within 5–10 years). Thus, it is possible that participants judged the cost associated with some of the more highly valued research questions to exceed available donor resources. Furthermore, despite our attempt to reach (via email) a large group of stakeholders with a wide range of backgrounds and roles, the participants of this exercise were skewed toward academics, which may bias results; however, those who did respond had substantial experience providing technical support to programs.

In summary, the study team found it possible to apply the CHNRI methodology to develop a list of research questions required to improve the implementation of this important and cost-effective nutritional intervention in LMIC populations. These research gaps need to be urgently addressed. Given the simple nature and relatively low cost of this exercise, this research prioritization exercise could be repeated periodically as new information becomes available.

## Disclaimer

N.D. is a UNICEF staff member. The opinions expressed in the documents included in this article are those of the authors and do not necessarily reflect the policies or views of UNICEF.
