# Supplementary material for: Setting research priorities on multiple micronutrient supplementation in pregnancy
Source: Ann N Y Acad Sci. 2019 Nov 6:10.1111/nyas.14267. doi: 10.1111/nyas.14267 (PMC7186835; doi:10.1111/nyas.14267)
Supplement: Supplementary file 1 [file ANYAS-2019-NYAS-14267-s1.docx]

**Table S1.** Research questions ranked according to the final weighted research priority score (RPS)

| **Research priority score, weighted (%)** | **Rank** | **Question** | **Domain** | **Sub-domain** |
| --- | --- | --- | --- | --- |
| 83.5 | 1 | What strategies (cash transfers, easier ANC access, free MMS, pharmacy vouchers, quality service delivery, mass media, social and behavior change communication interventions, SMS text messages, etc.) can best increase ANC attendance and adherence to MMS, including in hard to reach populations? | Delivery | Coverage |
| 82.9 | 2 | What limited set of biomarkers of nutritional status (e.g., hemoglobin) and their cut-offs can be used to identify populations that will benefit from prenatal MMS? | Description | Assessment |
| 82.0 | 3 | If MMS were continued through lactation, are there additional benefits for the mother and child (e.g., reduced mortality, infection, improved development, etc.)? | Discovery | Impact |
| 81.1 | 4 | Can community workers help identify pregnancies in the first trimester and facilitate timely ANC attendance that leads to an earlier initiation of MMS? | Delivery | Coverage |
| 79.6 | 5 | What is the burden of micronutrient deficiencies among pregnant women? | Description | Prevalence |
| 78.2 | 6 | What field friendly methods can be used to assess multiple micronutrient deficiencies among pregnant women? (contrast all methods along cost effectiveness, invasiveness, training requirements) | Description | Assessment |
| 76.8 | 7 | Which essential micronutrients (e.g., biomarkers or intake) beyond iron should be routinely monitored for pregnant women? | Description | Assessment |
| 75.2 | 8 | What are the most effective counselling strategies about the benefits of MMS in pregnancy that lead to increased adherence to the MMS regimen? | Delivery | Adherence |
| 75.0 | 9 | Are MMS in pregnancy effective in women with low intakes of energy and protein? | Discovery | Impact |
| 74.2 | 10 | What MMS dosage (timing and duration) should be recommended in prepregnancy and pregnancy to achieve maximum adherence and benefits on outcomes? | Development | Implementation |
| 73.9 | 11 | Can human-centered design principles (focused on the needs, contexts, behaviors, and emotions of the people) be used to increase the effectiveness of behavior-change programs and increase adherence to prenatal MMS? | Delivery | Adherence |
| 73.2 | 12 | To what extent do MMS benefit maternal health (not just anemia or pregnancy outcomes)? | Discovery | Impact |
| 73.2 | 13 | How can a policy framework be strengthened within a country to ensure availability of MMS supplements? | Development | Implementation |
| 73.1 | 14 | What is the most cost-effective packaging of MMS (i.e. blister packs or bulk packaging; 30-, 90-, or 180-count bottles, etc.) that will optimize both cost and adherence, without adversely affecting ANC attendance? | Delivery | Packaging |
| 72.9 | 15 | What are the sufficient and cost-effective training options when switching from IFA to MMS, e.g., (1) standard one-time in-service training; (2) enhanced training, supervision and coaching delivered routinely every few weeks for an initial period; and (3) enhanced training plus community engagement and promotion? | Delivery | Training |
| 72.3 | 16 | In pregnant women taking MMS who develop iron deficiency anemia, what is the ideal amount and duration of additional iron supplements? | Development | Dosage |
| 72.1 | 17 | What is the optimal dose of iron (30 versus 60 mg) in MMS to achieve maximum benefits on maternal and birth outcomes? Does it vary by context, population prevalence of anemia and dosage of other nutrients (e.g., vitamin C)? | Development | Dosage |
| 70.9 | 18 | What data commonly available in national surveys can be used to identify populations that will benefit from prenatal MMS? | Description | Prevalence |
| 70.7 | 19 | What indicators can be measured through routine health information systems to best monitor program performance in relation to MMS delivery during pregnancy (through ANC contacts)? | Delivery | Coverage |
| 70.4 | 20 | To what extent do infections blunt the impact of prenatal MMS in preventing anemia? | Discovery | Impact |
| 69.6 | 21 | What are the predictive risk factors of micronutrient deficiencies among pregnant women? | Description | Prevalence |
| 68.9 | 22 | Would outcomes be further improved by the addition of choline to MMS, especially with regard to child development?  What would be the cost implications? | Discovery | Formulation |
| 68.9 | 23 | Would pregnancy outcomes be further improved by the addition of calcium to MMS, given WHO recommendations for calcium supplementation during pregnancy to reduce risk of pre-eclampsia?  How would this affect adherence, costs, and stability (given iron and calcium interaction)? | Discovery | Formulation |
| 68.5 | 24 | Would birth outcomes be further improved by the addition of n-3 LC-PUFA to MMS, given a recent Cochrane meta-analysis showing reduction in preterm delivery with n-3 LC-PUFA supplementation? What would be the cost implications? | Discovery | Formulation |
| 68.4 | 25 | How can implementation research be most efficiently conducted (time and cost) to improve adherence to prenatal MMS? | Delivery | Adherence |
| 68.0 | 26 | What is the effectiveness, in terms of availability, acceptability, and adherence of public versus private sector MMS distribution? | Delivery | Coverage |
| 67.5 | 27 | Is fortification of food staples or ensuring intake of fortified foods (such as lipid-based nutrient supplements), better than providing MMS at scale, on maternal and birth outcomes? | Discovery | Formulation |
| 66.2 | 28 | Is Selenium deficiency independently associated with prematurity and small-for-gestational age? | Discovery | Impact |
| 66.2 | 29 | Would outcomes be further improved by the addition of magnesium to MMS?  What would be the implications on adherence and costs? | Discovery | Formulation |
| 65.2 | 30 | Are there subpopulations at risk of adverse outcomes with MMS, such as stillbirths or perinatal asphyxia? | Discovery | Impact |
| 65.0 | 31 | When compared to UNIMMAP, are there more cost-effective formulations? | Development | Dosage |
| 61.9 | 32 | What is the most appropriate dosage for each micronutrient, other than iron? | Discovery | Formulation |
| 55.6 | 33 | How does micronutrient status during early life development relate to adult onset of noncommunicable diseases? | Description | Prevalence |
| 54.7 | 34 | Why is MMS more successful in preventing infant mortality in female infants than in male infants? | Discovery | Impact |
| 52.1 | 35 | What is the marginal cost and marginal benefit of adding each vitamin/mineral to MMS? | Development | Dosage |

Note: The questions are color coded by type of domain: yellow for “description,” green for “delivery,” orange for “development,” and blue for “discovery”
